# Antifungal Activity of Propyl Disulfide from Neem (*Azadirachta indica*) in Vapor and Agar Diffusion Assays against Anthracnose Pathogens (*Colletotrichum gloeosporioides* and *Colletotrichum acutatum*) in Mango Fruit

**DOI:** 10.3390/microorganisms9040839

**Published:** 2021-04-14

**Authors:** Muhammad Rafiullah Khan, Vanee Chonhenchob, Chongxing Huang, Panitee Suwanamornlert

**Affiliations:** 1 School of Light Industry and Food Engineering, Guangxi University, Nanning 530004, China; khan87@gxu.edu.cn; 2 Department of Packaging and Materials Technology, Kasetsart University, Bangkok 10900, Thailand; 3 Center for Advanced Studies for Agriculture and Food, KU Institute for Advanced Studies, Kasetsart University (CASAF, NRU-KU, Thailand), Bangkok 10900, Thailand; 4 College of Integrative Medicine, Dhurakij Pundit University, Bangkok 10210, Thailand; panitee.tip@dpu.ac.th

**Keywords:** natural plant extract, antimicrobial, microorganisms, neem, propyl disulfide, mango, postharvest decay, anthracnose

## Abstract

Microorganisms causing anthracnose diseases have a medium to a high level of resistance to the existing fungicides. This study aimed to investigate neem plant extract (propyl disulfide, PD) as an alternative to the current fungicides against mango’s anthracnose. Microorganisms were isolated from decayed mango and identified as *Colletotrichum gloeosporioides* and *Colletotrichum acutatum.* Next, a pathogenicity test was conducted and after fulfilling Koch’s postulates, fungi were reisolated from these symptomatic fruits and we thus obtained pure cultures. Then, different concentrations of PD were used against these fungi in vapor and agar diffusion assays. Ethanol and distilled water were served as control treatments. PD significantly (*p* ≤ 0.05) inhibited more of the mycelial growth of these fungi than both controls. The antifungal activity of PD increased with increasing concentrations. The vapor diffusion assay was more effective in inhibiting the mycelial growth of these fungi than the agar diffusion assay. A good fit (*R*^2^, 0.950) of the experimental data in the Gompertz growth model and a significant difference in the model parameters, i.e., lag phase (λ), stationary phase (*A*) and mycelial growth rate, further showed the antifungal efficacy of PD. Therefore, PD could be the best antimicrobial compound against a wide range of microorganisms.

## 1. Introduction

Mango (*Mangifera indica* L.) is an important tropical fruit due to its favorable flavor, rich nutrition and high marketing value. However, this fruit is highly susceptible to various pathogens, leading to quality deterioration and significant economic losses. A variety of *Colletotrichum* spp. are responsible for causing anthracnose in various fruits, with a causal agent of anthracnose in mango being *Colletotrichum gloeosporioides*. Pathogens infect immature mango fruit as a latent infection, and the lesions progressively appear after storage and ripening [1,2].

Anthracnose can be controlled to a great extent by the applications of synthetic fungicides such as benomyl, carbendazim, thiabendazole, prochloraz, dithiocarbamate and azoles. Nonetheless, numerous adverse effects are associated with synthetic fungicides, such as toxicity, environmental pollution and potential risks to human health [2,3,4,5]. These negative effects have increased consumer awareness of food safety. Furthermore, the development of middle to high levels of resistance of pathogens to the existing fungicides has compelled the researchers to find other alternatives.

Recently natural plant extracts have become of great interest to researchers due to their volatile nature, aroma, antioxidant and antimicrobial properties [6,7]. Further, plant disease management with plant essential oils has been recognized as one of the best approaches [8]. A considerable number of essential oils exhibited antimicrobial activities against different pathogens [9,10]. Plant extracts such as aroma compounds (*trans*-cinnamaldehyde, citral, and phenylacetaldehyde [11], *Mentha piperita* L. essential oil (MPEO) [12], volatile organic compounds [2], essential oil from *Cymbopogon citratus* (D.C. ex Nees) Stapf [13], and essential oil extracted from *Cympopogon citratus* and *Lippia rehmannii* [14] have suppressed anthracnose in mango fruit. Feng et al. [3] evaluated the antifungal activity of camptothecin (a naturally occurring quinoline alkaloid having a high pesticidal activity) against *C. gloeosporioides* causing anthracnose disease in mango. Dessalegn et al. [15] evaluated the plant defense-inducing chemicals (PDIC) combined with postharvest treatments with inorganic salts and hot water to prevent mango’s anthracnose.

Neem plant (*Azadirachta indica*) is a rich repository of more than 300 primary and secondary metabolites and is a powerful natural pesticide [16]. Many bioactive compounds have been isolated from neem plants and have antifungal, antibacterial, antioxidant and other biological properties [17,18,19]. Thus far, neem extracts are primarily used in pharmaceutical or traditional medicines; few studies are reported against postharvest pathogens. For example, the extract of neem leaf was used to inhibit the growth of *Botrytis cinerea, Aspergillus flavus* and *Aspergillus niger* fungi causing anthracnose in wild mango [20] and *Aspergillus viridae, Penicillium digitatum* and *Rhizopus* sp. causing anthracnose in tomato [21].

Propyl disulfide is one of the active compounds in neem seed and is a potent pesticide in grain storage. Recently, we worked on the antifungal activity of propyl disulfide on mango fruits against *Lasiodiplodia theobromae* and *Neofusicoccum parvum,* causing the stem-end rot in mango fruit. Propyl disulfide was much more effective in controlling the growth of these pathogens [22]. Interestingly, when the Gompertz growth model was applied, the data were a good fit in the model and propyl disulfide had a positive effect on the stationary phase and lag phase of the microbial growth [22].

As anthracnose is one of the major diseases in postharvested fruits, we continued our study to work on propyl disulfide from neem against the major pathogens causing anthracnose in mango with the aim to find a potential leading compound for fungicide development. A pathogenicity test was conducted to confirm the major pathogens of mango causing anthracnose. Furthermore, a growth model was also applied to see the accuracy and good fit of the experimental and theoretical results.

## 2. Materials and Methods

### 2.1. Plant Materials and Chemicals

**Figure 1:**
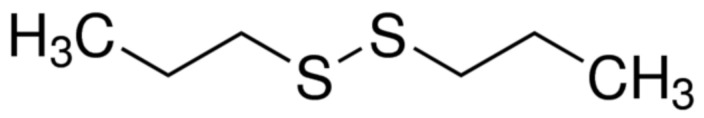
Structure of propyl disulfide.

**Figure 2:**
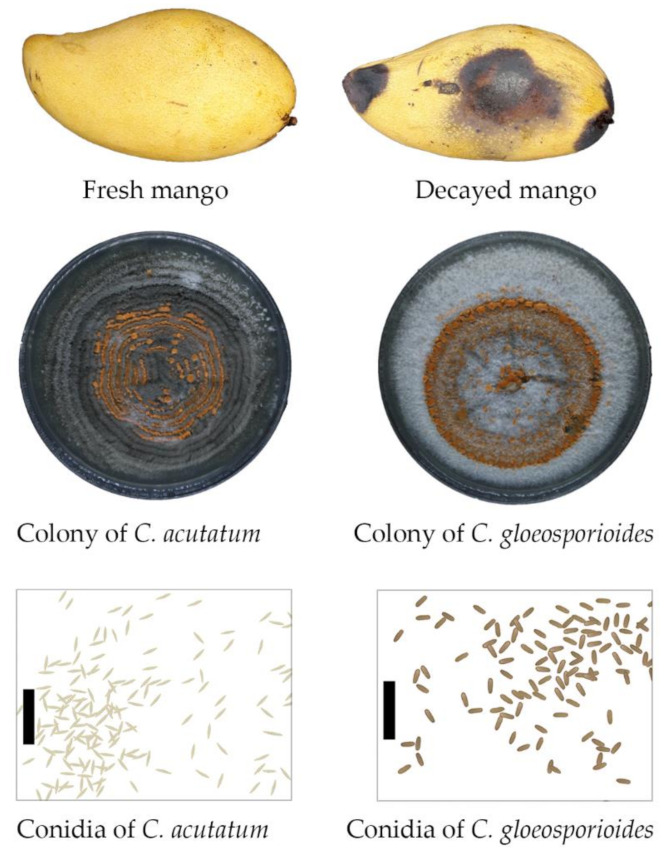
*C. acutatum* and *C. gloeosporioides* isolated from the decayed mangoes and their respective colonies and conidia. Bar = 10 µm.

Propyl disulfide (purity ≥ 97%, FG, oil phase) (Figure 1), a volatile antifungal compound of neem extract (*Azadirachta indica*) was obtained from Sigma-Aldrich (St Louis, MO, USA). Ripe mangoes (*Mangifera indica*) brought from the local market were stored in the laboratory at ambient temperature for decay symptoms, as shown in Figure 2.

### 2.2. Isolation and Identification of Fungi

Fungi were isolated and identified according to the method of Khan et al. [22]. Briefly, fresh and healthy mangoes were washed with clean tap water to remove any soil debris, the surface sterilized with NaOCl (1%) and stored at ambient temperature for fungal growth. Small pieces of active lesions (triplicate) from decayed mango (Figure 2) were separated with the help of a sterile blade and washed with NaOCl (1%) for 3 min, rinsed with sterile distilled water, dried and transferred to Petri dishes containing potato dextrose agar (PDA) (one piece per plate). The Petri dishes were sealed with Parafilm and incubated for 7 days at ambient temperature (26 ± 3 °C). Upon the mycelial growth, a small colony was reisolated and transferred to a new Petri dishes, incubated and checked visually and morphologically under the stereo and compound microscopes. This process was repeated several times to obtain pure cultures and based on the colony and conidial features; these fungi were identified as *C. gloeosporioides* and *C. acutatum*, the causal microbes of anthracnose.

### 2.3. Pathogenicity Test

A pathogenicity test was conducted to establish Koch’s postulates following the protocols of Khan et al. [22] and Wanjiku et al. [23]. Fresh and healthy mangoes were washed with clean tap water to remove any soil debris, the surface sterilized with NaOCl (1%) and kept in trays. A sterile cork borer (1 cm diameter) was used to wound the fruit and mycelial disc obtained from the edge of actively growing pure cultures (7 days old, obtained in Section 2.2) was placed on the wound. Trays were covered with plastic film, moisturized and stored at room temperature of 26 ± 3 °C. Finally, after the pathogenicity test, reisolation from the symptomatic fruit was carried out, the fungal colonies and conidial features were compared to the original isolates (Figure 2). Actively growing pure cultures were transferred to PDA slants, screw test tubes (plugged with cotton) or in Petri dishes (wrapped with parafilm to reduce drying) containing agar medium. After the establishment of culture, these were stored in room temperature 26 ± 3 °C or refrigerated temperature of 4 °C by following the protocol of Karabıçak et al. (2016) [24] as stock cultures and were checked periodically for contamination and desiccation. Before each experiment, fungi were grown in fresh PDA and incubated at room temperature.

### 2.4. Antifungal Activity of Propyl Disulfide

A two-fold serial dilution method was used to prepare different concentrations by dissolving 25, 50, 100, 200, 400 and 800 g L^−1^ of PD in absolute ethanol (AR grade; Merck; Darmstadt, Germany). Antifungal activity of the PD was conducted in both vapor and agar diffusion assays [22,25].

For the vapor phase assay, a sterile filter paper (47 mm diameter) was fixed with the Petri dish’s inside lid. Then, a 5 mm mycelium plug taken from actively grown pure culture was positioned upside down in the center of a 90 mm Petri dish containing PDA. From each concentration of PD, 50 µL was placed on the attached sterile filter paper. Filter paper with ethanol and sterile distilled water were used as control treatments. Petri dishes were immediately sealed with Parafilm to prevent vapor loss and all plates were stored at ambient temperature 26 ± 3 °C until mycelial growth reached the margin of the Petri dish in control treatments. Each concentration was conducted in five replicates.

Similarly, 50 µL from each concentration was used in agar diffusion assay; here, the PD was mixed with PDA in Petri dish. After solidification, a 5 mm of mycelium plug taken from actively grown pure culture was kept upside down in each Petri dish and sealed immediately with Parafilm. Ethanol and sterile distilled water were incorporated in PDA as control treatments. All the plates were incubated at ambient temperature 26 ± 3 °C until the mycelia reached to the edge of Petri dish in control treatments. Each treatment was conducted in five replicates. The radial mycelial growth was measured (in mm) with a Vernier caliper in two perpendiculars and the mean diameter was obtained every 24 h.

A minimum inhibitory concentration (MIC) was defined as the lowest concentration of the antifungal compound that prevents visible mycelial growth of the fungi. In this study, MIC of PD was taken into account with respect to its different concentrations against the fungal radial growth (mm) and compared to distilled water and ethanol following the protocol of Khan et al. [22] and Suwanamornlert et al. [25]. Percent growth inhibition (PI) of each concentration of the propyl disulfide was calculated with the following formula [3,22].

(1) $$PI=\frac{[\left(C-I\right)-\left(T-I\right)}{\left(C-I\right)}\times 100\;$$

where *C* = mycelial growth diameter of the control; *I* = initial mycelial plug diameter (5 mm) and *T* = mycelial growth diameter of the pathogen exposed to propyl disulfide at each concentration.

### 2.5. Gompertz Model for Fungal Growth

Gompertz growth model was applied to find the mango fungal growth model and its parameters [22,26].

(2) $$ln(Dt/D0)=A\;exp\left\{-exp\left[\left(\frac{Vm\;.e}{A}\right)\left(\lambda -t\right)+1\right]\right\}\;$$

where *Dt* (mm); the average colony diameter at time t, *D*0 (mm); average colony diameter at the initial time, *A*; maximum colony diameter at stationary phase, *Vm;* maximum growth rate (1/time), λ; the lag phase (time) and *e* = exp. (1). The model parameters were measured by regression analysis using Microsoft Excel. The value of mean square error (MSE) and the coefficient of determination (*R*^2^) were used to determine the goodness-of-fit of the model.

### 2.6. Statistical Analysis

Data were subjected to analysis of variance with Duncan’s new multiple range test at a significance level of *p* ≤ 0.05 using SPSS version 16 software (SPSS Inc., Chicago, IL, USA).

## 3. Results

### 3.1. Anthracnose Rot and Characteristics of Isolated Fungi

The symptoms of anthracnose caused by *Colletotrichum* spp. were dark brown and black lesions (Figure 2). The cultural and morphological investigations revealed that *C. acutatum* and *C. gloeosporioides* were causing anthracnose disease in mango fruit. These fungi were differentiated by their colony colors, growth rates, the presence or absence of a teleomorph and conidial morphologies. Mycelium of *C. acutatum* were white to light grey and orange, growth was relatively slow and reached the edge of Petri dish in about 14 days. Conidia of *C. acutatum* were fusiform and cylindrical with a narrow end. On the other hand, the mycelium of *C. gloeosporioides* was whitish to orange, becoming deep orange or dark gray with time, having a regular or an irregular margin with both submerged and aerial topography. The mycelial growth rate of *C. gloeosporioides* was relatively fast and reached the edge of the Petri dish in about 10 days. *C. gloeosporioides* mostly produced hyaline, one-celled, ovoid to oblong, slightly curved or dumbbell-shaped conidia with thickheaded and round end with well developed hyaline conidiophores (Figure 2).

### 3.2. Antifungal Activity of Propyl Disulfide

**Figure 3:**
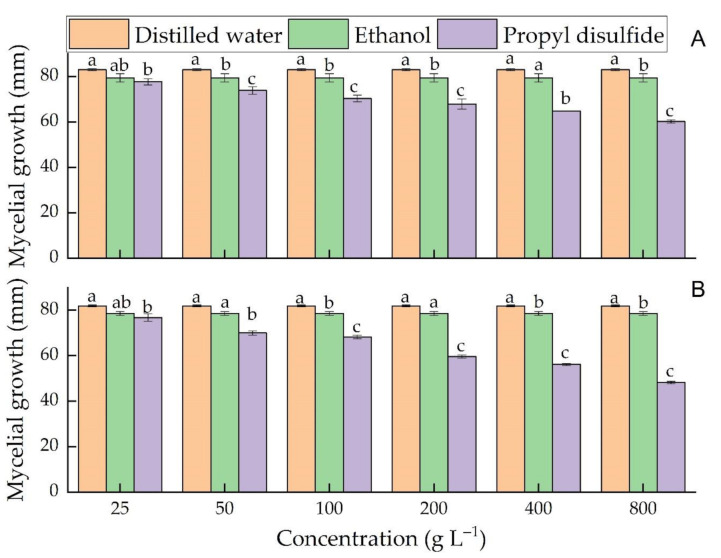
Effects of different concentrations of propyl disulfide in the vapor phase assay on the mycelial growth of *C. acutatum* at day 14 (**A**) and *C. gloeosporioides* at day 10 (**B**). Different small letters in each concentration represent a significant difference among the treatments. The mycelial growth presented is the mean of five replicates ± SD at *p* ≤ 0.05.

In both vapor and agar diffusion assays, the antifungal activity of PD increased with its concentration. In vapor phase assay, both controls and PD (25 g L^−1^) were nonsignificantly different, while at 50 g L^−1^ and above, PD significantly inhibited the mycelium growth of *C. acutatum*. Among the control treatments, ethanol was significantly more effective than distilled water (Figure 3A). Regarding the *C. gloeosporioides,* both controls and PD (25 g L^−1^ and 50 g L^−1^) were nonsignificantly different, while at 100 g L^−1^ and above, PD significantly (*p* ≤ 0.05) inhibited the mycelium growth of *C. gloeosporioides* (Figure 3B).

**Figure 4:**
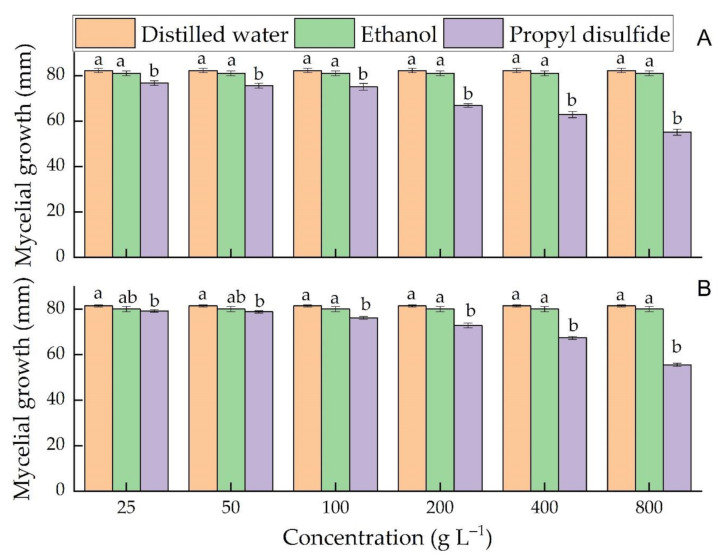
Effects of different concentrations of propyl disulfide in the agar diffusion assay on the mycelial growth of *C. acutatum* at day 14 (**A**) and *C. gloeosporioides* at day 10 (**B**). Different small letters in each concentration represent a significant difference among the treatments. The mycelial growth presented is the mean of five replicates ± SD at *p* ≤ 0.05.

Similarly, in the agar diffusion assay, PD was significantly effective than both controls at all concentrations in *C. acutatum* and 100 g L^−1^ and above in the case of *C. gloeosporioides*. Again, in both tested fungi, the antifungal efficacy of PD increased with increasing its concentrations (Figure 4A,B). However, in agar diffusion assay, no significant difference was found in distilled water and ethanol in *C. acutatum* and *C. gloeosporioides* (Figure 4A,B).

Interestingly, in the vapor phase assay, the percent growth inhibition of PD was higher (*p* ≤ 0.05) for *C. gloeosporioides* than *C. acutatum* (Table 1), while in the agar diffusion assay, the percent growth inhibition of PD was significantly higher (*p* ≤ 0.05) for *C. acutatum* than *C. gloeosporioides* (Table 2). The minimum inhibitory concentration of PD was higher than 800 g L^−1^ in both fungi. However, both fungi responded differently when exposed to the different concentrations of PD in both the vapor and agar diffusion assays (Table 1 and Table 2).

### 3.3. Gompertz Model for Fungal Growth

**Figure 5:**
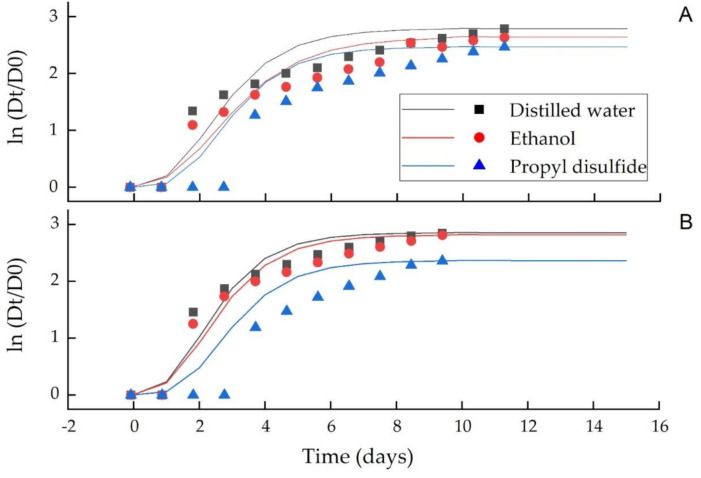
Effect of PD in the vapor diffusion assay on the growth of *C. acutatum* (**A**) and *C. gloeosporioides* (**B**). The mycelial growth presented is the mean of five replicates ± SD at *p* ≤ 0.05. Dt is the average colony diameter at time t and D0 is the average colony diameter at initial time, fitted (distilled water, ethanol and propyl disulfide) with the modified Gompertz model.

**Figure 6:**
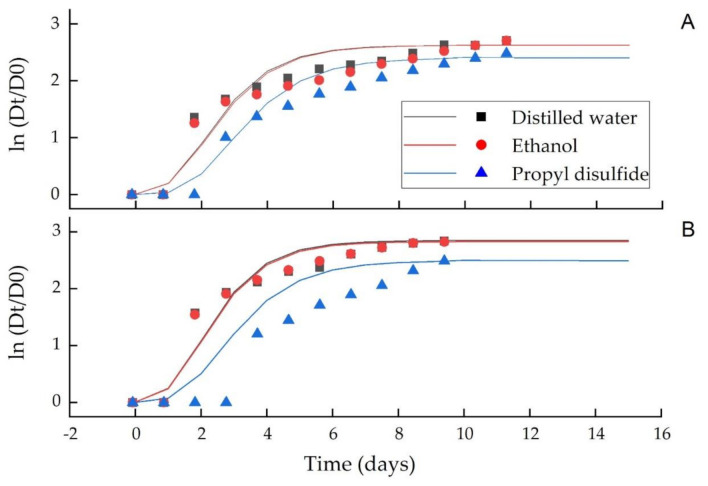
Effect of PD in the agar diffusion assay on the growth of *C. acutatum* (**A**) and *C. gloeosporioides* (**B**). The mycelial growth presented is the mean of five replicates ± SD at *p* ≤ 0.05. Dt is the average colony diameter at time t and D0 is the average colony diameter at initial time, fitted (distilled water, ethanol and propyl disulfide) with the modified Gompertz model.

Figure 5 and Figure 6 show the growth curves of *C. acutatum* and *C. gloeosporioides* against the distilled water, ethanol and PD, while their model parameters (*A*, *Vm*, λ) are summarized in Table 3 and Table 4 for the vapor and agar diffusion assays, respectively. The model *R*^2^, 0.95 showed a good fit of the experimental data to the model equation. A decrease in the mycelial growth during the stationary phase (*A*) indicated a higher antifungal PD activity than distilled water and ethanol. In the vapor phase assay, the maximum growth rate (*V_m_*) was significantly (*p* ≤ 0.05) different in all treatments for both *C. acutatum* and *C. gloeosporioides* (Table 3), whereas in agar diffusion assay, the maximum growth rate (*V_m_*) was significantly (*p* ≤ 0.05) different in PD than distilled water and ethanol for both *C. acutatum* and *C. gloeosporioides* (Table 4). Propyl disulfide also significantly (*p* ≤ 0.05) enhanced the lag phase (λ) of both fungi from 1.32 to 1.53 d as compared to 0.89 to 0.99 d in ethanol and 0.88 to 0.96 d in distilled water in the vapor and agar diffusion assays, respectively (Table 3 and Table 4).

## 4. Discussion

Anthracnose, caused by *Collectrotrichium* spp. is a major postharvest disease of mangoes that results in substantial economic losses. In addition, in light of the development of medium to high levels of resistance of these pathogens to the existing fungicides, the present work is the first report on the anthracnose rot while using an active volatile compound (PD) from neem to develop a potential new fungicide. *Colletotrichum* spp. infects the mango fruit by producing appressoria from germinating spores that penetrate into the surface of the fruits. The fungus usually attacks the fruit in the early stages and typically remains dormant until the fruit ripens. Upon ripening, dark spots develop, enlarging to form lesions that may amalgamate and cover almost the fruit’s entire surface [2]. In this study, pathogens were identified based on their cultural, morphological and conidial structures such as shape, size and colony color. Similar characteristics were previously reported by [27,28] in mango fruit. Pathogenicity test results revealed that the inoculated mango fruits showed the anthracnose symptoms and the causal agents of the fruit rot were reisolated, cultured and identified as *C. gloeosporioides* and *C. acutatum*, thus fulfilling Koch’s postulates.

Propyl disulfide was effective in inhibiting the mycelial growth of these fungi. Furthermore, the slower mycelial growth in ethanol than the distilled water suggested its antimicrobial efficacy against mango’s anthracnose. Antimicrobial activity of ethanol against anthracnose was found in guava [29]. The mycelial growth inhibition was higher in the vapor phase assay than in the agar phase assay, which was probably due to the accumulation of the antifungal compound’s vapors and its slow release in the Petri dish’s headspace. This shows that fumigation, which slowly releases the active compound, makes the fungi more susceptible to treatment than the direct contact method. This effect was also found in *L. theobromae* and *N. parvum* of stem-end rot pathogens when exposed to propyl disulfide [22]. In another study conducted by our research group, [25] it was found that the vapor diffusion assay of thymol, carvacrol and trans-cinnamaldehyde had stronger antifungal activities than agar diffusion assay on the longan pathogens, i.e., *Lasiodiplodia* spp., *Phomopsis* spp., *Pestalotiopsis* spp. and *Geotrichum candidum*.

Compared to our previous work [22], in the present study, we found that PD was more prominent in preventing the stem-end rot pathogens than anthracnose. In stem-end rot, the 800 g L^−1^ PD growth inhibition was 71.70% and 70.66% in vapor phase assay and 63.47% and 68.15% in the direct contact assay (agar diffusion assay) for *L. theobromae and N. parvum,* respectively [22]. While in the current study, the percent growth inhibition of 800 g L^−1^ PD was 29.23% and 43.87% in vapor phase assay and 35.19 and 33.93% in agar diffusion assay for *C. acutatum* and *C. gloeosporioides,* respectively (Table 1 and Table 2). In present study an interesting finding was that in vapor phase assay, the percent growth inhibition of the 800 g L^−1^ PD was higher (*p* ≤ 0.05) for *C. gloeosporioides* (43.87%) than *C. acutatum* (29.23%) (Table 1), while in agar diffusion assay, the percent growth inhibition of 800 g L^−1^ PD was higher (*p* ≤ 0.05) for *C. acutatum* (35.19%) than *C. gloeosporioides* (33.93%) (Table 2). This also confirmed that each fungus responded differently when exposed to the different concentrations of PD. However, in all cases, the percentage growth inhibition of the PD against the tested fungi increased with increasing the concentration (Table 1 and Table 2). This suggests that antifungal activity of PD is a dose-dependent activity, as previously found by [20] that 60% aqueous solution of neem leaf extract was more effective than 40% and 20% in inhibiting the mycelial growth of *Botrytis cinerea, Aspergillus flavus* and *Aspergillus niger* in mango fruit. It also indicates that the growth reduction of the major pathogens isolated from mango is attributed to PD rather than ethanol. Mycelial growth of *C. gloeosporioides* was also inhibited by using aroma compounds, i.e., *trans-cinnamaldehyde*, citral and phenylacetaldehyde [11]. Feng et al. [3] reported an effective inhibition of *C. gloeosporioides* in mango while using camptothecin (CPT-1), a naturally occurring quinoline alkaloid with significant cantineoplastic and pesticidal activities. Mycelial growth of *C. gloeosporioides* and other pathogens such as *Alternaria citri*, *Diaporthe citri*, *Geotrichum citri-aurantii*, *Penicillium digitatum*, *Penicillium italicum* causing postharvest decay in citrus fruit were inhibited by the use of pinocembrin-7-glucoside (P7G) extracted from Ficus hirta Vahl. fruit [30].

The current study revealed that MIC value of the PD compound was higher than 800 g L^−1^ in both vapor and direct contact assays. However, the antifungal activity varied with the fungi; as can be seen in both vapor and agar diffusion assays, that stem end rot pathogens (*L. theobromae* and *N. parvum*) were more vulnerable [22] than anthracnose pathogens (*C. acutatum and C. gloeosporiodies*) (Table 1 and Table 2). This shows that the antifungal effect of the active compound depends on the target microorganisms.

The antifungal activity of PD could be attributed to the sulfur compound as sulfur compounds are well-known for microbial growth prevention. According to Koul [31], the physiological toxicity and behavioural interaction makes PD a potential grain protectant against the insect system. The author fumigated grain pest, i.e., *Sitophilus oryzae* and *Tribolium castaneum,* with 1, 10 or 20 mg L^−1^ of propyl disulfide and diallyl disulfide and stated that the high deterrence index induced by propyl disulfide shows its higher potential effectiveness than diallyl disulfide. Lyer and Williamson [32] accredited the antifungal properties of neem extracts to inhibit protease activity of dermatophytes induced by the neem extract. Ramos et al. [33] stated that antiadhesive mechanism of the neem extract on the cell surface’s hydrophobicity and biofilm formation could affect the colonization of the *C. albicans*. Zhong-hui et al. [34] and Okemo et al. [35] suggested that the neem extract’s killing ability against different pathogenic microorganisms depends on extraction time, concentration and cell wall constituents. Kumar and Kudachikar [11] stated that the possible antifungal mechanisms of natural plant extracts against pathogens could be attributed to the disruption of membrane integrity and cellular components’ leakage. Zakawa et al. [20] used neem leaf extracts and prevented the mycelial growth of *Botrytis cinerea, Aspergillus flavus* and *Aspergillus niger* fungi causing postharvest rot in mango. Suleiman [21] also used neem leaf extract and prevented the mycelial growth of *Aspergillus viridae, Penicillium digitatum* and *Rhizopus* spp. causing anthracnose postharvest rot in tomato.

A good fit (*R*^2^, 0.950 of the experimental data in the model equation and a significant difference in the model parameters, i.e., lag phase (λ), stationary phase (*A*) and mycelial growth rate, further showed the greater antifungal efficacy of PD than distilled water and ethanol. Therefore, it can be stated that this compound could also be a useful fumigant material against a wide range of pathogens.

## 5. Conclusions

Anthracnose is one of the major diseases in mango fruit, causing considerable economic losses. To some extent, the disease could be successfully prevented by using the existing fungicides; however, the medium to high levels of resistance of the pathogens to the current fungicides have compelled researchers to find other suitable alternatives. Therefore, the present research was carried out to test PD’s fungicidal effect from neem plants’ seeds against anthracnose in the mango. Anthracnose causing fungi were first isolated and subcultured many times to obtain a pure culture. A pathogenicity test was conducted to confirm the causal agents to fulfill the Koch’s postulates, and major pathogens were identified as *C. acutatum* and *C. gloeosporioides* causing anthracnose in mango. The results revealed that PD more effectively inhibited the mycelial growth of *C. acutatum* and *C. gloeosporioides* than distilled water and ethanol. Furthermore, a significant difference in the Gompertz growth model parameters and a good fit of the model suggested that PD could be the best alternative to the existing fungicides in controlling anthracnose in mango and other fruits and could decrease the huge economic losses in the fresh produce industry.

## Figures and Tables

**Table 1:** Percentage of mycelial growth inhibition (%) of major pathogens isolated from mango exposed to various concentrations of propyl disulfide in vapor phase compared to control stored at ambient temperature (26 ± 3 °C).

Concentrations (g L^−1^)	*C. acutatum* (Day 14)	*C. gloeosporioides* (Day 10)
800	29.23 ^aB^ (0.88)	43.87 ^aA^ (1.70)
400	23.41 ^bB^ (2.89)	33.53 ^bA^ (1.75)
200	19.34 ^cB^ (2.06)	28.93 ^cA^ (1.93)
100	17.96 ^cA^ (0.78)	17.73 ^dA^ (0.55)
50	11.74 ^dB^ (2.54)	15.51 ^dA^ (1.70)
25	5.26 ^eA^ (2.00)	6.63 ^eA^ (2.39)
MIC *	˃80	˃80

Different small letters within the column and capital letters within the row represent a significant difference between different concentrations and within fungi at each concentration, respectively. Means are separated by the DMRT test (*p* ≤ 0.05). Each point is the average of five replicates; standard deviation (SD) is given in parentheses. MIC * = minimum inhibitory concentration.

**Table 2:** Mycelial growth inhibition (%) of major pathogens isolated from mango exposed to various concentrations of propyl disulfide incorporated in the potato dextrose agar (PDA) medium (agar diffusion assay) at ambient temperature (26 ± 3 °C).

Concentrations (g L^−1^)	*C. acutatum* (Day 14)	*C. gloeosporioides* (Day 10)
800	35.19 ^aA^ (2.84)	33.93 ^aA^ (1.10)
400	25.15 ^bA^ (1.07)	18.43 ^bB^ (2.13)
200	19.93 ^cA^ (1.50)	11.36 ^cB^ (2.18)
100	9.33 ^dA^ (1.09)	6.95 ^dB^ (1.73)
50	8.64 ^dA^ (1.71)	3.44 ^eB^ (1.15)
25	7.15 ^dA^ (2.66)	2.95 ^eB^ (0.82)
MIC *	˃80	˃80

Different small letters within the column and capital letters within the row represent a significant difference between different concentrations and within fungi at each concentration, respectively. Means are separated by the DMRT test (*p* < 0.05). Each point is the average of five replicates; standard deviation (SD) is given in parentheses. MIC * = minimum inhibitory concentration.

**Table 3:** Modified Gompertz model parameters for vapor diffusion assay.

Antifungal Agents	*C. acutatum*					*C. gloeosporioides*				
	A (mm)	$v_{m}$ (1/d)	λ (d)	MSE	*R* ^2^	A (mm)	$v_{m}$ (1/d)	λ (d)	MSE	*R* ^2^
Distilled water	2.79 ^a^ (0.01)	0.81 ^a^ (0.01)	0.96 ^b^ (0.01)	0.091	0.905	2.86 ^a^ (0.01)	0.94 ^a^ (0.00)	0.91 ^b^ (0.00)	0.041	0.949
Ethanol	2.64 ^b^ (0.03)	0.66 ^c^ (0.01)	0.99 ^b^ (0.00)	0.077	0.911	2.82 ^a^ (0.02)	0.87 ^b^ (0.02)	0.94 ^b^ (0.01)	0.044	0.938
Propyl disulfide	2.47 ^c^ (0.02)	0.76 ^b^ (0.02)	1.32 ^a^ (0.12)	0.244	0.750	2.36 ^b^ (0.02)	0.74 ^c^ (0.02)	1.38 ^a^ (0.09)	0.189	0.763

Different superscript letters within each column are significantly different (*p* ≤ 0.05). The standard deviation (SD) is given in parentheses below the average.

**Table 4:** Modified Gompertz model parameters for agar diffusion assay.

Antifungal Agents	*C. acutatum*					*C. gloeosporioides*				
	A (mm)	$v_{m}$ (1/d)	λ (d)	MSE	*R* ^2^	A (mm)	$v_{m}$ (1/d)	λ (d)	MSE	*R* ^2^
Distilled water	2.63 ^a^ (0.01)	0.84 ^a^ (0.01)	0.93 ^b^ (0.01)	0.056	0.939	2.85 ^a^ (0.02)	0.97 ^a^ (0.01)	0.88 ^b^ (0.02)	0.052	0.934
Ethanol	2.63 ^a^ (0.01)	0.82 ^a^ (0.01)	0.94 ^b^ (0.00)	0.084	0.903	2.83 ^a^ (0.02)	0.96 ^a^ (0.01)	0.89 ^b^ (0.00)	0.039	0.952
Propyl disulfide	2.40 ^b^ (0.01)	0.69 ^b^ (0.01)	1.53 ^a^ (0.17)	0.064	0.924	2.49 ^b^ (0.01)	0.72 ^b^ (0.01)	1.33 ^a^ (0.00)	0.225	0.722

Different superscript letters within each column are significantly different (*p* ≤ 0.05). The standard deviation (SD) is given in parentheses below the average.

## Data Availability

Not applicable.
